# Vaccination with dendritic cells loaded with HIV-1 lipopeptides elicits broad T cell immunity and control of viral load in HIV infected patients

**DOI:** 10.1186/1742-4690-9-S2-P328

**Published:** 2012-09-13

**Authors:** Y Levy, R Thiébaut, M Montes, C Lacabaratz, L Sloan, S Perusat, C Harrod, C Boucherie, S Zurawski, L Richert, G Chêne, J Banchereau, K Palucka

**Affiliations:** 1 INSERM U955-Université Paris Est, Créteil, France; 2 INSERM, U897, France Univ Bordeaux Segalen, Bordeaux, France; 3 INSERM U899-Baylor Institute for Immunology Research, Dallas, TX, USA; 4 Baylor Institute for Immunology Research, Dallas, TX, USA; 5 INSERM U897, Bordeaux, France

## Background

The DALIA trial tested the hypothesis that immunization with HIV peptide loaded Dendritic Cells (DC) may improve HIV immune responses and help to contain viral replication.

## Methods

19 pts with CD4 >500 cells/m3 and HIV RNA <50 cp/ml under HAART received at W0, 4, 8 and 12 ex-vivo generated IFN-α DC loaded with HIV-1 lipopeptides. Analytical treatment interruption (ATI) was conducted from W24. HAART resumption regardless of the reason and CD4 <350 cells/mm3 (or <25%) were considered as end points. HIV-specific immunity was evaluated at baseline, W16, and W48 using: i) ex vivo IFN-γ ELISPOT; ii) intra cellular staining; iii) multiplex analysis. PBMCs were stimulated with HIV peptide pools. Student t-test and Wilcoxon signed-rank tests were used with estimation of the False Discovery Rates (FDR) for controlling test multiplicity.

## Results

Vaccine regimen was well tolerated. Following ATI, all pts experienced a viral rebound in 14 days in median (IQR 8-27). Median highest observed VL (peak) was 5 (4.28-5.23) log10 cp/ml. Three patients resumed HAART and eight had CD4 <350 cells/mm3. Median (IQR) SFU/106 PBMC rose from 186 (140-670) at baseline to 761 (470-1154) and 1878 (1102-4443) at W16 and 48, respectively. At the same time points the breadth of the response (nb of peptide pools) increased from 1 (1-3) to 4 (2-5) (P=.009) and 6 (3-7) (P=.008). % of polyfunctional CD4+ (> 2 cytokines among: IFN-γ,TNF-α,IL-2) increased from 0.026% (w-4) to 0.32% (w16) (P=.002). Respective % of CD8+ were 0.26% and 0.35% (P=.005). Production of IL-2, IFN-g, IL-21, IL-13, IL-17 increased significantly at W16 (FDR<.05). An inverse correlation was found between the peak of VL and % of polyfunctional CD4+ (r=-0.63, FDR=.007), production of IL-2 (r=-0.67, FDR=.006), IFN-g (r=-0.58, FDR=.01), IL-21 (r=-0.66, FDR=0.006) and IL-13 (r=-0.78, FDR=.001).

## Conclusion

DC vaccination elicited polyfunctional HIV-specific responses associated with a reduced peak viral load following ATI.

